# Phenomenon of declining blood pressure in elderly - high systolic levels are undervalued with Korotkoff method

**DOI:** 10.1186/1471-2318-11-57

**Published:** 2011-10-03

**Authors:** Arkadiusz Siennicki-Lantz, Sölve Elmståhl

**Affiliations:** 1 Division of Geriatric Medicine, Skane University Hospital in Malmö, Lund University, SWEDEN

## Abstract

**Background:**

Systolic blood pressure (SBP) decline has been reported in octogenarians. The aim was to study if it could be observed while measuring SBP with two methods: Korotkoff (K-BP) and Strain-Gauge-Finger-Pletysmography (SG-BP), and which of them were more reliable in expressing vascular burden.

**Methods:**

A cohort of 703 men from a population of Malmö, Sweden, were included in "Men born in 1914-study" and followed-up at ages: 68 and 81 years. 176 survivors were examined with K-BP and SG-BP at both ages, and 104 of them with Ambulatory Blood Pressure at age 81/82. Ankle Brachial Index (ABI) was measured on both occasions, and Carotid Ultrasound at age 81.

**Results:**

From age 68 to 81, mean K-BP decreased in the cohort with mean 8.3 mmHg, while SG-BP increased with 13.4 mmHg. K-BP decreased in 55% and SG-BP in 31% of the subjects. At age 81, K-BP was lower than SG-BP in 72% of subjects, and correlated to high K-BP at age 68 (r = --.22; p < .05). SG-BP at age 81 was correlated with mean ambulatory 24-h SBP (r = .480; p < .0001), daytime SBP (r = .416; p < .0001), nighttime SBP (r = .395; p < .0001), and daytime and nighttime Pulse Pressure (r = .452; p < .0001 and r = .386; p < .0001). KB-BP correlated moderately only with nighttime SBP (r = .198; p = .044), and daytime and nightime pulse pressure (r = .225; p = .021 and r = .264; p = .007). Increasing SG-BP from age 68 to 81, but not K-BP, correlated with: 24-h, daytime and nighttime SBP, and mean daytime and nighttime Pulse Pressure. Increasing SG-BP was also predicted by high B-glucose and low ABI at age 68, and correlated with carotid stenosis and low ABI age 81, and the grade of ABI decrease over 13 years.

**Conclusion:**

In contrast to K-BP, values of SG-BP in octogenarians strongly correlated with Ambulatory Blood Pressure. The SG-BP decline in the last decade was rare, and increasing SG-BP better than K-BP reflected advanced atherosclerosis. It should be aware, that K-BP underdetected 46% of subjects with SG-BP equal/higher than 140 mmHg at age 81, which may lead to biased associations with risk factors due to differential misclassification by age.

## Background

Several studies have reported that in aging, blood pressure (BP) takes a shape of inverted parabola with initial BP-increase up to a seventh decade, and thereafter a subsequent decline [1,2]. BP decline was correlated with: a number of years before dementia onset [3,4], high initial BP level [5], shorter survival [6-8], cognitive decline [9,10] and dementia [11-14]. Different explanations for these corellates have been proposed, based on an observation that patients who died demented were characterized by a low BP and thin left ventricle posterior wall [15]. Cognitive impairment was suggested to be an effect of heart failure combined with hypotension [16,17]. On the other side, degeneration of brain could be responsible for improper autoregulation and lead to a lower BP, which secondary could increase brain sensitivity on the episodic BP variations [18].

In healthy hypertensive elderly, attempts to treat high BP gave positive results concerning the rate of stroke, heart failure, cardiovascular death and dementia [19,20]. In all mentioned studies, BP was measured using brachial cuff and auscultation by Korotkoff method, as an established office measurement method. The auscultatory Korotkoff technique, compared to intra-arterial measurement, tends however to give lower systolic values [21]. On the other hand, in elderly patients systolic BP tends to be overestimated, which could be revealed by the positive Osler's sign [22]. However, Osler's sign is not as frequent (11% of octogenerians) [23], and the true pseudohypertension is observed in only 2/3 of the Osler-positive elderly.

To avoid the pitfalls connected with auscultation and to increase reliability of BP measurement in labolatories, a strain gauge technique has been developed - where BP is recorded objectively by a curve [24]. This method is widely used e.g. for peripheral pressure measurements in arms, legs, ankles and toes. In this method, a pressure cuff is placed on the arm, like in the Korotkoff method, but peripheral flow is not detected by auscultation, but by using a pulse detector on a finger during the cuff deflation.

Concerning the possible pitfalls during blood pressure measurement using Korotkoff technique in the elderly, we aimed to study if aging-related changes in BP could be observed in the same extent by Korotkoff as Strain gauge technique, and which of them was best correlated to Ambulatory Blood Pressure Monitoring. Thanks the opportunity to examine BP with both Korotkoff and strain gauge technique in the cohort of elderly men twice during their life, we could compare the time-change in BP with vascular risk factors, as well as with established markers of vascular disease. The null hypothesis was that there would be no difference between age-related BP decline examined with these two methods.

## Methods

### Population

A prospective population study "Men born in 1914" has been in progress since 1968. 809 men born in even months in 1914 in Malmö- Sweden were invited, and 703 of them took part in the first examination. At first follow-up, at age 68, 500 agreed to participate in a new examination. At the second follow-up, 281 men who were at life and reached 81 years, were invited 185 agreed to participate (66%). BP measurements according to the protocol below were done in 176 subjects. Additional examinations were done at age 68, including laboratory analyses and circulation in lower extremities estimated by ankle-brachial pressure index (ABI). At age 81, except repeated medical examination, a medical history was retrieved from a questionnaire, spouses and/or relatives, and carotid ultrasound was performed, as well as ABI was estimated again.

### Blood pressure measurement

Upper right arm systolic and diastolic BP was measured sphygmomanometrically at age 68 and at age 81, after 15 min of rest, using a calibrated mercury manometer and a standard rubber cuff (12 × 35 cm). For obese patients, 15 cm cuff were used. All measurements at age 81 were made by one and the same physician. Additionally, at both follow-ups, BP has been measured by Strain Gauge Pletysmography Technique (SG). This method has been mainly used for labolatory measurements, e.g. for peripheral pressure measurements in arms, legs, ankles and toes, for the last four decades [24]. The validity of the method was calculated by comparing it to the intraarterial measurement [25]. The occluding cuff was placed on the same as in the Korotkoff method. Instead of auscultation of the brachial artery, finger blood flow was continuously recorded by mercury-in-silastic strain gauge pulse sensor placed on the proximal phalanx of the first digit. Signal was amplified by Wheatstone bridge for recording the resistence of the strain gauges, and joined to a pressure transducer (Siemens-Elema EMT 746 with amplifier EMT 311) to record cuff pressures. Arithmetic average of two recordnings was used. The arm cuff pressure was released 1-2mmHg per second. At a certain arm cuff pressure, when blood escaped under the cuff, the change of electric impedance of strained mercury occured. This was observed as an increase of the strain gauge tracing and the registered BP at this moment was taken to be the systolic BP. The same technique was used at age 68 and 81 at the Dept of Clinical Physiology, Malmö University Hospital, Malmö, Sweden, in a standardized way, performed daily by experienced technicians and data interpreted by trained physicians. Hypertension was defined as auscultatory systolic and diastolic brachial BP ≥ 160 mmHg or ≥ 90 mmHg respectively, or medication for hypertension. These hypertension criteria have been used and were valid until World Health Organisation drawn up new ones at 1999 [26]. All study subjects were monitored and treated during their lifetime according to these hypertension criteria, and they were also chosen for statistical analysis.

### Peripheral arterial circulation

Ankle BP was estimated by placing a cuff at the ankle level and using Doppler signal on tibial posterior artery or dorsal foot artery. Reference pressure in the arm was calculated using SG-BP method as above. The arithmetic average of duplicate recordnings in supine position was used. Ankle-brachial pressure index (ABI) was calculated by dividing the ankle systolic BP with the highest upper arm systolic BP.

### Carotid Duplex ultrasonography

The examination of carotid arteries was performed using computed sonography system (Acuson XP 10, Acuson, USA) with a 7 MHz B-mode real-time linear scanner. A 5 MHz pulsed, color-coded Doppler was used to localize areas with high flow velocities in the internal carotid artery, and the maximum flow velocity (m/s) was measured.

### Ambulatory blood pressure monitoring

Ambulatory blood pressure monitoring was performed during the first year after the last follow-u, using Micro AM Recorder, Model KI5600 (Kontron Instruments; SpA, Milan, Italy). Readings at 20 min intervals during a day (from 06.20 AM to 09.40 PM) and at 60 min intervals at night (from 10.00 PM to 06.00 AM) were performed. Monitoring was performed in patient's private environment without specific advices regarding physical activity. The ambulatory BP data were obtained primary by the auscultatory method and in case of failure repeated with oscillometric method. The accuracy of KI5600 was confirmed by a simultaneous measurement with a standard mercury sphygmomanometer, and accepted if they were within 10 mmHg of standard method. The exclusion of patients was made according to the quality criteria: deficit in measurement time intervals at least 6 h accumulated during a daytime or more than 3 h accumulated at nighttime, or more than 3 h consecutively during a daytime or at least 2 h consecutively during a nighttime. Pulse pressure daytime and nighttime, an established vascular disease marker, was estimated as a mean of individual difference values between SBP and DBP, and used for analysis. 136 subjects agreed to participate, but 104 fullfiled the quality criteria and were chosen to statistical anlysis.

### Statistical analysis

Summary values are expressed as mean ± standard deviation. Correlation analyses were performed using Pearson correlation test. Differences in vascular risk factors/markers were calculated with Mann-Whitney Rank Sum Test. All data analysis has been performed using SPSS (SPSS Inc., Chicago, IL, USA) statistical package. A two tailed *P *value of less than 0.05 was considered statistically significant. Local ethical committee at Lund University accepted the study, and informed consent was obtained from all participants.

## Results

The baseline data in 176 study subjects of the study are presented in table 1 and include blood pressure measurments with both Korotkoff method (K-BP), Strain gauge method (SG-BP) and Ambulatory Blood Pressure Monitoring, together with vascular risk factors and vascular markers at age 68 and 81 years.

**Table 1:** Baseline data in 176 study subjects of blood pressure measurmenets with both Korotkoff method (K-BP), Strain gauge method (SG-BP) and Ambulatory Blood Pressure Monitoring, as well as of vascular risk factors and vascular markers at age 68 and 81 years

Baseline data	Mean (SD)
*At age 68:*	
K-BP systolic, mmHg	151.9 (20.7)
K-BP diastolic	97.9 (10.2)
SG-BP systolic	147.0 (18.5)
Alcohol consumption (g/week)	83.3 (113.6)
Body Mass Index	24.9 (2.9)
B-glucose	4.99 (.079)
P-Cholesterol	5.98 (.092)
ABI (ankle-brachial pressure index), right	1.04 (.14)
ABI, left	1.04 (.16)
*At age 81*:	
K-BP systolic, mmHg	143.6 (15.1)
K-BP diastolic	82.3 (6.2)
SG-BP systolic	160.1 (23.9)
Carotid stenosis, right (%; median)	35.0 (0-75)
Carotid stenosis, left (%; median)	35.0 (0-90)
ABI, right	.097 (.19)
ABI, left	.095 (.20)
*Ambulatory Blood Pressure*^1^	
24-h SBP, mmHg	128.8 (11.7)
24-h DBP	73.5 (10.1)
Daytime SBP	131.1 (12.0)
Daytime SD-SBP (mean standard deviation SBP)	13.1 (3.05)
Daytime DBP	75.5 (10.4)
Daytime SD-DBP	10.1 (2.9)
Nighttime SBP	120.1 (10.1)
Nighttime SD-SBP	11.7 (4.2)
Nighttime DBP	67.6 (10.9)
Nighttime SD-DBP	9.5 (3.4)
Pulse pressure day, mmHg	55.6 (8.2)
Pulse pressure night	53.4 (8.1)

^1 ^Valid for 104 subjects

### Difference between BP-values measured by Korotkoff and Strain-Gauge method

**Figure 1:**
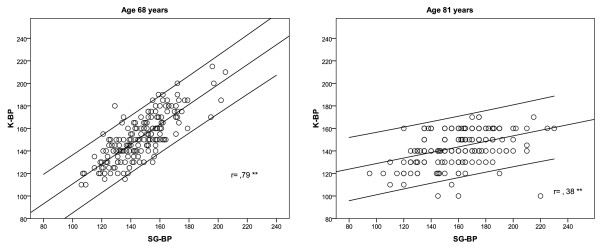
**Correlation between Strain Gauge (SG-BP) and Korotkoff blood pressure (K-BP) measurement in the same subjects at age 68 and at age 81**. Regression line with 95% confidence intervals.

At age 68, an arithmetic difference between values of K-BP and SG-BP in each subject was calculated and the mean value in the whole cohort was 5.2 mmHg (SD 12.9). K-BP and SG-BP measurements were strongly correlated to each other (r = .79; p < .0001). In that age, 33% of subjects had lower K-BP than SG-BP. At age 81, a mean of arithmetic differences between K-BP and SG-BP was -16.5 mmHg (SD 23.0), and the correlation between those measurements was significant (r = .38; p < .0001) (Figure 1). As much as 71.6% of subjects had lower K-BP than SG-BP at that time.

### Time-course of BP measured by different methods

**Figure 2:**
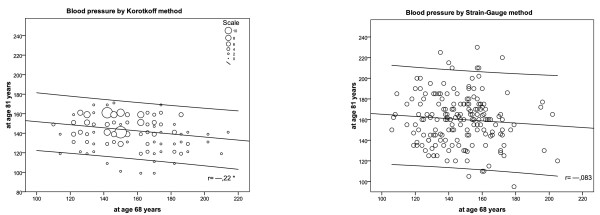
**Correlation between Korotkoff blood pressure (K-BP) at age 68 and at age 81 (left), and between Strain Gauge blood pressure (SG-BP) at age 68 and at age 81 (right)**. Regression lines with 95% confidence intervals.

During the follow-up of 13 years, K-BP of the whole cohort decreased with mean 8.3 mmHg (SD 28.2). Contrary, SG-BP changed over time with an increase with mean 13.4 mmHg (SD 31.5). At an individual level, K-BP decreased over time in 55.1% of the subjects, while SG-BP decreased only in 31.3% of the subjects. K-BP at age 68 correlated negatively with K-BP at age 81 (r = --.22; *p = .003*), but no correlation was observed between SG-BP values at 68 and 81 (r = --.083; *p = .274*) (Figure 2).

### Which method accounts for the difference between the two BP measurements?

**Figure 3:**
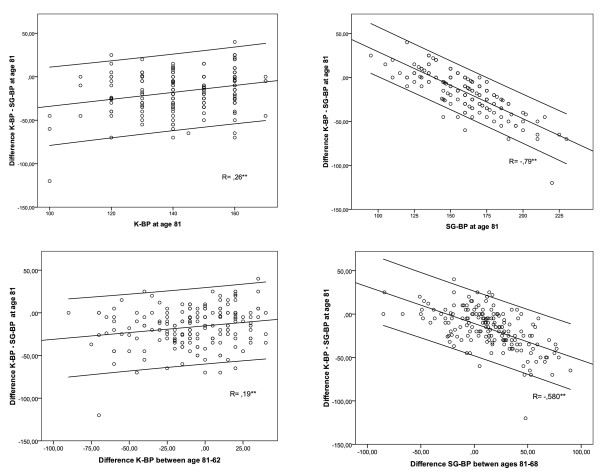
**Correlation between the arithmetic difference Korotkoff - Strain Gauge blood pressure values (K-BP - SG-BP) at age 81 and individual blood pressure variables at age 81 (upper row), and blood pressure differences during follow-up period (bottom row)**.

An arithmetic difference between K-BP and SG-BP in each individual at age 81 correlated positively with K-BP at age 81 (r = .263; p < .000) and negatively with SG-BP at age 81 (r = --.794; p < .000) (Figure 3; upper row). It also correlated positively with a difference in K-BP between ages 81-68 (r = .194; p = .01) and strongly negatively with a difference in SG-BP (r = --.58; p < .000) (Figure 3; nether row). An arithmetic difference between K-BP and SG-BP was not correlated to individual values of K-BP or SG-BP at age 68.

### Which method predicts or reflects vascular disease?

Vascular risk factors and markers of vascular disease has been examined both at age 68 and 81, and correlated to time-course of BP (arithmetic difference between age 81-68).

Fasting b-glucose levels at age 68 predicted increasing SG-BP, but not K-BP (Table 2). Low values of ABI at age 68 predicted both increasing SG-BP and K-BP. At the last follow-up, ABI and carotid stenosis estimated at age 81 expressed increasing SG-BP, but not K-BP (Table 2). The aggravating peripheral arterial disease, expressed as a negative arithmetic difference between ABI at age 81 and 68, had the strongest correlation to increasing SG-BP over follow-up-time (Figure 2).

**Table 2:** Coefficients of correlation between vascular risk factors/markers of atherosclerosis (rows), ambulatory blood pressure and time-course of blood pressure measured by Korotkoff (K-BP) or Strain-Gauge method (SG-BP) (columns)

	Difference in K-BP between age 81 - 68	Difference in SG-BP between age 81 - 68
*At age 68:*		
Alcohol consumption	-.061	-.060
BMI	.029	.099
B-glucose level	-.015	.161*
P-cholesterol level	-.018	-.097
ABI, right	.099	.156
ABI, left	.175*	.156*
*At age 81*:		
Carotid stenosis, right	-.066	.029
Carotid stenosis, left	.133	.155*
ABI, right	.056	-.114
ABI, left	-.071	-.187*
Difference ABI right, age 81-68	-.083	-.160*
Difference ABI left, age 81-68	-.162*	-.239*
*Ambulatory Blood Pressure*^1^		
24-h SBP	.011	.239*
Daytime SBP	.045	.222*
Daytime SD-SBP	-.055	.298*
Nighttime SBP	.040	.264*
Nighttime SD-SBP	.150	.124
Pulse pressure day	.112	.372**
Pulse pressure night	.139	.330**

* p < 0, 05;** p < 0, 0001; ^1 ^Valid for 104 subjects;

### Which method is best correlated with Ambulatory Blood Pressure?

SG-BP at age 81 was correlated with mean 24-h SBP (r = .480; p < .0001), daytime SBP (r = .416 ; p < .0001), nighttime SBP (r = .395; p < .0001), mean daytime variability (SD-SBP) (.417; p < .0001), and mean daytime and nighttime pulse pressure (r = .452; p < .0001 and r = .386; p < .0001 respectively). Systolic KB-BP at age 82 did not correlate with either 24-h SBP, daytime SBP, or SBP variability, but correlated with nighttime SBP (r = .198; p = .044), and daytime and nightime pulse pressure (r = .225; p = .021 and r = .264; p = .007 respectively).

Increasing SG-BP from age 68 to 81 (i.e. arithmetic difference SG-BP age 81-68) correlated with 24-h SBP, daytime SBP, nighttime SBP, mean daytime variability (SD-SBP), and mean daytime and nighttime pulse pressure (Table 2). Time-change in KB-BP did not correlate with either 24-h SBP, daytime or nighttime SBP, SD-SBP, or pulse pressure.

### Clinical consequences

Applying modern hypertension criteria, at age 81, 137 study subjects had SG-BP equal or higher than 140 mmHg, while only 74 (54%) if measured with K-BP method (Table 3). Hypertension stage 2, with BP equal or higher than 160 mmHg, was found in 84 subjects with SG-BP method, but only 4 (5%) of them reached that level using K-GP method (Table 3).

**Table 3:** Distribution of systolic hypertension at age 81, defined as values above 140 mmHg (part A), and above 160 mmHg (part B), estimated by both Korotkoff method (K-BP) and by Strain-Gauge Pletysmography (SG-BP)

**A**.		SG-BP > 140 mmHg	
		No (n = 39; 12%)	Yes (n = 137; 88%)
**K-BP > 140 mmHg**	No	29 (74%)	63 (46%)
	Yes	10 (26%)	74 (54%)
**B**.		**SG-BP > 160 mmHg**	
		No (n = 92; 52%)	Yes (n = 84; 48%)

**K-BP > 160 mmHg**	No	92 (100%)	80 (95%)
	Yes	0 (0%)	4 (5%)

### Drop-out analysis

Drop-out analysis compared pressure data between those study subjects who died between follow-ups or did not agree to participate, mainly due to the poor health (307 subjects) and those who reached the last follow up at age 81 (176 subjects). Systolic and diastolic K-BP did not differ significantly between those groups (SBP mean ± SD: 154 ± 22 and 151 ± 21 respectively, *p *=, 329; DBP: 93 ± 11 and 92 ± 10 respectively, *p *=, 428). However, SG-BP was significantly higher in the drop-out subgroup than in those who participated in the follow-up at 81 (152 ± 21 vs. 147 ± 18; *p *=, 006).

## Discussion

In our study, K-BP and SG-BP measurements correlated with each other in both ages, but the correlation was weaker at age 81. At that age, the vast majority of subjects had lower K-BP than SG-BP. During the 13-years-long observation period, blood pressure decreased in a large part of the sample, but the proportion of subjects with declining pressure was nearly double when measured by Korotkoff method, compared to SG-BP. An important finding was that, paradoxically, those subjects who had highest K-BP at age 68, had also lowest K-BP values 13 years later. That was not observed using SG-BP method. The difference between K-BP and SG-BP at age 81 was mainly explained by growing SG-BP, and to a lesser extent by decreasing K-BP.

Ambulatory blood pressure monitoring, even if performed only in the two thirds of the sample, could be used as a reference point for the two methods. SG-BP correlated strongly with 24-h, both daytime and nighttime SBP values, and reflected by higher SBP-variability. Contrary, K-BP was only correlated and in a moderate grade with nocturnal SBP. It suggests that SG-BP is a much more reliable method in octogenarians to estimate high systolic BP levels.

The clinical validity of SG-BP, contrary to K-BP, was also tested by correlating their time-change with markers of vascular disease and vascular risk factors. Growing SG-BP, from 68 to 81, in the best way reflected the established markers of atherosclerosis at age 81: low ABI, high daytime and nighttime Pulse Pressure, and carotid stenosis. Aggravating atherosclerosis, i.e. decreasing ABI during follow-up, was also best correlated with increasing SG-BP level.

The role of values from the drop-out group, i.e. subjects who died before reaching age 81, had higher SG-BP than K-BP at age 68, which suggests that SG-BP reflects in a better way the cardiovascular burdon which lead to early death in the study. The data on the relationship between increasing SG-BP and aggravated atherosclerotic process emphasize that BP decline could be overestimated by Korotkoff method.

The validity of auscultatory Korotkoff method was examined in the 1950-70-ties, showing generally lower systolic K-BP than intra-arterial BP with -0, 4 to -24 mmHg and a range from -70 to + 48 mmHg [25]. In a population aged 18-73, auscultatory SBP was on the average 8, 8 mmHg below that of intra-arterial SBP. As in our study, Nielsen observed that the auscultatory vs. intra-arterial systolic BP-artefact increased with increasing intra-arterial pressure [25]. Contrary to our study, no correlation between age and the measure artefact could be demonstrated, however, a mentioned study did not include octogenarians. Similar difference was observed between oscilometric and intra-arterial pressure [27]. The K-BP vs. SG-BP examination artefact, mostly seen with increasing BP in our study, was previously observed using intra-arterial measurement as a golden standard [28,29]. Similar discrepancy exists between oscillometric and intra-arterial BP methods in critically ill obese patients with hypertension [30]. This seems to be explained not by the inability to record the first audible Korotkoff sound, but by the increasing "critical closing pressure" with the increasing level of BP.

Measurement of SG-BP was introduced in 1960-ies for detection of peripheral arterial disease in legs and toes [31]. Its validity was tested in subjects 18-50 years old, and the average intra-arterial SBP was 7 mmHg above SG-BP with a sensor placed on the finger, and 8 mmHg with a sensor placed on the toe [25]. In that study, in young and middle-aged sample, a mean difference between intra-arterial vs. brachial K-BP and brachial SG-BP was similar, which reflects the validity of the latter method. SG-BP was found to be reproducible and correlated to auscultation in finger, ankle, and arm in middle-aged subjects [24,25]. Pressure gradients between arm and legs have been calculated in healthy populations sample, and in those with with vascular risk factors, when the method was widely used to examine peripheral circulation [32].

What could be the possible explanation of the underestimated BP with Korotkoff method in the oldest old? It should not be possible that vascular pressure wave reaches distal vessels before the brachial artery itself, and that we detect a pulse signal in the finger before we hear the Korotkoff sound. If we try to explain that artefact, we must understand, that in aging, hypertension is a combination of arterial stiffening, early wave reflection and central BP. In the elderly, the amplitude of the brachial and radial pulse pressure is nearly equal to that in ascending aorta due to the back-reflection of the forward pressure wave, transmitted from the central aorta by high-resistance arterioles. The pulse wave is then amplified toward the periphery at any point of the impedance discontinuity, such as arterial branches. Therefore, the pressure waveform in the brachial artery is a sum of the forward pressure wave generated by the heart and the forward reflected pressure wave from the body. In the elderly, the reflected pressure wave returns during early systole in ascending aorta, through the fact that arterial stiffness augments the aortic pulse pressure, similarly as the brachial. It leads to a low amplification. In younger subjects, the reflected wave doesn't augment aortic and brachial pulse pressure, because it returns during late systole or early diastole in the artery and cannot contribute to high pulse pressure. It leads instead to higher amplification [33]. The phenomenon of lower amplification of the wave could result in a lower first Korotkoff sound in the elderly, though at the same time, the pulse wave expands the tissue in the finger. It has also been shown, that most of the energy contained in the external pulse recorded during cuff deflation is below the audible range. The wideband of external pulse wave, recorded from the distal portion of the cuff, changes in a characteristic manner as cuff pressure is reduced from above systolic, to below diastolic, pressure. Normal subjects exhibited a Korotkoff first sound, in which the early systolic peak was larger than the late systolic peak, and all subjects who exhibited other variations of first Korotkoff sound, were patients with had a known cardiovascular disease [34].

Another mechanism for measurement pitfall could be the pulse wave velocity, which increases with age, atherosclerosis, and BP, and is dependent upon vessel elasticity, as well as an independent risk factor for cardiovascular disease [33]. It could be measured as a time between the onset of the depolarization on electrocardiogram (Q) and as point of detection of the last Korotkoff sound (K) at the level of the brachial artery during cuff deflation, corresponding to diastolic blood pressure (D), called QKD interval. A reduction in mean QKD interval has been found with age and hypertension, reflecting the recognized higher pulse wave. The slopes of the plots of QKD interval, versus BP, were small indicating lower influence of BP-change on pulse wave velocity in patients with stiffer arteries [35]. Combining this fact with evidence of lower pulse wave amplification in the elderly, a high pulse wave velocity can be earlier detected by the finger pulse sensor than by an auscultation [36].

There can be three sources of error in the indirect measurement of BP: observer bias, faulty equipment, and failure to standardize the techniques. At both follow-ups, K-BP was examined by experienced physicians, aware of the measurements pitfalls in elderly, and all measurements at age 81 were made by one and the same physician. SG-BP was a standardized method from the same labolatory. Selective mortality of individuals at high risk has not influenced on the results. However, 105 invited subjects chose not to participate, mainly for health reasons, expressing higher frequency of vascular disease as their SG-BP was higher 13 years earlier. Changing exposure to risk factors and changing therapy regime during the follow-up could be another source of bias.

## Conclusions

Both cross-sectional and longitudinal data of this study point out a significant underestimation of BP with Korotkoff technique and underdetection of hypertension in men over 80-ty, leading to biased associations with risk factors due to differential misclassification by age. Systolic blood pressure values on Ambulatory Blood Pressure Monitoring were highly correlated with SG-BP, and not with K-BP, at age 81. Peripheral vascular disease, and its dynamics, were stronger related to increasing SG-BP during the 13 years follow-up. The clinical relevance of these observations seems to be substantial since only 54% of subjects with systolic BP level equal or above 140 mmHg, according to Strain gauge method, were detected with the Korotkoff method. This phenomenon should be made aware for clinicians and researches treating octogenarians or designing future treatment studies.

## Competing interests

The authors declare that they have no competing interests.

## Authors' contributions

ASL was involved with data analysis and manuscript preparation. SE was involved with data collection and manuscript preparation. All authors read and approved the final manuscript.

## Pre-publication history

The pre-publication history for this paper can be accessed here:

http://www.biomedcentral.com/1471-2318/11/57/prepub
